# Complex metabolic interactions between ovary, plasma, urine, and hair in ovarian cancer

**DOI:** 10.3389/fonc.2022.916375

**Published:** 2022-08-02

**Authors:** Xiaocui Zhong, Rui Ran, Shanhu Gao, Manlin Shi, Xian Shi, Fei Long, Yanqiu Zhou, Yang Yang, Xianglan Tang, Anping Lin, Wuyang He, Tinghe Yu, Ting-Li Han

**Affiliations:** ^1^ Department of Obstetrics and Gynaecology, The Second Affiliated Hospital of Chongqing Medical University, Chongqing, China; ^2^ State Key Laboratory of Ultrasound Engineering in Medicine Co-Founded by Chongqing and the Ministry of Science and Technology, School of Biomedical Engineering, Chongqing Medical University, Chongqing, China; ^3^ Department of Obstetrics, The First Affiliated Hospital of Chongqing Medical University, Chongqing, China; ^4^ Department of Oncology, The Second Affiliated Hospital of Chongqing Medical University, Chongqing, China; ^5^ Liggins Institute, The University of Auckland, Auckland, New Zealand

**Keywords:** metabolomics, ovarian cancer (OC), ovary, plasma, urine, hair

## Abstract

Ovarian cancer (OC) is the third most common malignant tumor of women accompanied by alteration of systemic metabolism, yet the underlying interactions between the local OC tissue and other system biofluids remain unclear. In this study, we recruited 17 OC patients, 16 benign ovarian tumor (BOT) patients, and 14 control patients to collect biological samples including ovary plasma, urine, and hair from the same patient. The metabolic features of samples were characterized using a global and targeted metabolic profiling strategy based on Gas chromatography-mass spectrometry (GC-MS). Principal component analysis (PCA) revealed that the metabolites display obvious differences in ovary tissue, plasma, and urine between OC and non-malignant groups but not in hair samples. The metabolic alterations in OC tissue included elevated glycolysis (lactic acid) and TCA cycle intermediates (malic acid, fumaric acid) were related to energy metabolism. Furthermore, the increased levels of glutathione and polyunsaturated fatty acids (linoleic acid) together with decreased levels of saturated fatty acid (palmitic acid) were observed, which might be associated with the anti-oxidative stress capability of cancer. Furthermore, how metabolite profile changes across differential biospecimens were compared in OC patients. Plasma and urine showed a lower concentration of amino acids (alanine, aspartic acid, glutamic acid, proline, leucine, and cysteine) than the malignant ovary. Plasma exhibited the highest concentrations of fatty acids (stearic acid, EPA, and arachidonic acid), while TCA cycle intermediates (succinic acid, citric acid, and malic acid) were most concentrated in the urine. In addition, five plasma metabolites and three urine metabolites showed the best specificity and sensitivity in differentiating the OC group from the control or BOT groups (AUC > 0.90) using machine learning modeling. Overall, this study provided further insight into different specimen metabolic characteristics between OC and non-malignant disease and identified the metabolic fluctuation across ovary and biofluids.

## Introduction

Ovarian cancer (OC) is the third most prevalent malignancy tumor among gynecological cancers worldwide with estimated 313,595 new cancer cases and 207,252 deaths annually in 2020 (1). OC is considered a silent killer because of rapid asymptomatic development and a lack of diagnostic approaches that often lead to poor prognosis (2, 3). At present, researchers do not understand its underlying pathogenesis, which makes the treatment outcome unsatisfactory (4, 5). Thus, it is necessary to understand the pathophysiology of OC and discover a more robust diagnostics tool.

Metabolic rewiring of cancer cells is crucial for cancer initiation, proliferation, and progression (6–8). Malignant tumors alter metabolites consumption, following generate molecular products such as metabolite byproducts and building blocks (9, 10). For example, according to the Warburg effect, tumors increase glucose consumption and secrete high quantities of lactic acid even in the presence of oxygen (11). In addition to glucose, other nutrients, including lipids and amino acids (glutamine, leucine, serine, etc.), have an increased uptake and consumption by cancer cells (12–14). Lipids can also serve as energy reservoirs for energy-carving malignant cells, alleviate cellular stress involved in the metastatic cascade, and resist chemotherapeutic treatments (15). Tumor metabolism can cause systemic metabolic changes according to the supply of oxygen and nutrients as well as the removal of waste products from blood vessels (16, 17). Nevertheless, the interaction between cancer tissue and systemic metabolism still remains unclear.

Metabolomics is the qualitative and quantitative analysis of low molecular weight metabolites (<1.5 kDa) detected within cells and a biological system (18). Metabolite profile is a measure of the precursors, intermediates, and products of metabolic pathways and, as such, is often recognized as more representative of the phenotypic state of a cell (19–22). Metabolomics has been utilized to investigate metabolic properties in OC (23–25). Hilvo et al. and colleagues reported that hydroxybutyric acid was accumulated in OC tissue. Also, the presence of epithelial-to-mesenchymal transition (EMT) gene expression, indicated a role for this metabolite changes in cancer cell invasion and migration (26). The low phospholipids and essential amino acids (citrulline) in the ovary were associated with less adaptive immune cell tumor infiltration and correlated with worse outcomes in OC patients (20). Liu et al. proposed that three metabolites (hexadecenoic acid, 23-lactone, and di-hydrothymine) with a lower concentration in the drug-resistant group have the potential to predict the prognosis of chemotherapy (27). Notably, there are numerous metabolomic researches on blood and urine in OC patients, but the majority of these studies were focused on biomarker discovery (26, 28). Some research reported that fatty acids (C16, C22), amino acids (histidine, tryptophan), and other organic compounds (kynurenine, L-carnitine) can be considered as serum biomarkers for OC (28–31). Urine is another commonly used biological sample in clinical practice. Several studies ascertained some metabolic biomarkers (succinic acid, fumaric acid, N-acetyl glutamine, etc.) for OC in urine (32, 33). On the other hand, the hair metabolite profile has advantages over other more transient biological samples such as urine and blood as it potentially provides longitudinal information of metabolite changes. However, there are no hair metabolome studies for OC. Despite a vast number of OC metabolomic studies, there is no single study integrating the differential biospecimens of ovary tissue, blood, urine, and hair, to investigate the systemic metabolic change of OC in a whole body.

In the present study, we employed a comprehensive gas chromatography-mass spectrometry (GC-MS) profiling to identify the altered metabolites between control, BOT, and OC using different specimens (ovary, plasma, urine, and hair) to uncover the metabolic interactions of local tumor tissue to those changes in the systemic metabolome.

## Materials and methods

### Participants and characteristics collection

The study was ethically approved by the Research Ethics Committee of the Second Affiliated Hospital of Chongqing Medical University, China (202164), and works in accordance with the Declaration of Helsinki. All participants were recruited from the Second Affiliated Hospital of Chongqing Medical University and signed the informed consent before enrolment in this study from July 2020 to June 2021. Patients with the following inclusion and exclusion criteria were enrolled as follows: 1) Participants with severe chronic diseases such as hypertension, diabetes, infectious disease, metabolic disorders, or a diagnosis with malignant other than ovarian cancer were excluded from this study to minimize recruitment bias; 2) control group (n=14) including uterine fibroids or endometrioma without any ovarian lesion; 3) benign ovarian tumors group (n=16) such as teratoma and ovarian cyst, and 4) ovarian cancer group (n=17) were diagnosed preoperatively on the serum markers, including Carbohydrate Antigen (CA125 > 35 U/mL) and Human Epididymis Protein (HE4 > 70 pmol/L in pre-menopausal patients, or HE4 >140 pmol/L in post-menopausal patients) (34), and the imaging modality of sonography for evaluation of an adnexal mass. The final diagnoses of those patients were confirmed by postoperative pathological examination. Subsequently, another three independent groups (control group =12, BOT =13, and OC =16) were also recruited as an external validation of urine samples with the same inclusion and exclusion criteria. The dietary recommendation was given to participants on the first inpatient day. Patient characteristics such as age, BMI, gravidity, and parity were collected.

### Sample collection and preservation

None of the patients received any therapy such as chemotherapy, radiotherapy, or surgery prior to sample collection. Plasma, urine, and hair specimens for each patient were collected on the same day prior to surgery. Whole blood was collected in ethylenediaminetetraacetic acid (EDTA)-containing tubes by trained nurses and the mid-stream urine was collected as the first pass urine in the morning. Both collected biofluids were centrifuged at a speed of 2300 g for 10 min at 4°C, transferred supernatant into a 1.5 ml cryopreservation tube, and stored at −80°C until metabolite extraction (35). The hair sample was cut 1.0 cm away from the scalp and stored in aluminum foil at 4°C. Ovarian (tumor) tissue was obtained immediately after surgery. All the samples were subsequently frozen within a half-hour in liquid nitrogen and followed by long-term storage at -80°C. The specimen ID was labeled with numbers without any patient’s personal information.

### Sample preparations for plasma, urine, hair, and tissue

All samples were processed with a standard operating procedure.150 µL aliquots of thawed plasma or urine were mixed with three internal standards (IS) [20 μL of d4-alanine (Sigma, USA, 10 mM), d5-phenylalanine (Sigma, USA, 10 mM), and d5-tryptophan (Sigma, USA, 10 mM)]. To precipitate protein from the plasma or urine samples, 400 µL of cold methanol was added, followed by freezing at - 20°C for 30 min. Then the supernatant was isolated by centrifugation at 12,000 rpm for 15 min at 4°C. The tissue sample was prepared by dissecting 30.00 ± 0.50 mg into new tubes. After adding three internal standards and 400 μL cold methanol, tissues were homogenized *via* TissueLyser II (QIAGEN, USA) and centrifuged (10,000g, 15 min, 4°C) to isolate the supernatant. The hair sample was washed with methanol and distilled water twice, then air-dried in a fume hood. 5.00mg ± 0.50 mg hair was prepared and added to three internal standards, and then incubated with 1 ml sodium hydroxide (1 M) at 54°C for 18 h. To precipitate the salt and protein, 1 ml of methanol was added to the hair extracts, followed by vortexing for 30 seconds, and centrifuged at 4000 g for 5 min. The supernatant was mixed with 50 µl of 4M NaOH. Then these mixtures underwent derivatization consecutively.

### Methyl chloroformate (MCF) derivatization and Gas Chromatography-Mass Spectrometry (GC-MS) analysis

All prepared extracts were chemically modified to lower their boiling point by MCF derivatization, based on the method published in Nature protocols (36). The volatile compounds were then separated by ZB-1701 GC capillary column (30 m × 250 μm id × 0.15 μm with 5 m guard column, Phenomenex, CA, USA) and detected by GC-MS (Agilent 7890B-5977A) with electron impact ionization *via* electron emission at 70 eV. The GC-MS parameters were operated following the procedure in previous research (37). The GC-MS inlet was set at 290°C with the pulsed splitless mode, 1 ml/min in the flow rate of the helium carrier. The temperature was controlled at 280°C, 230°C, and 150°C of auxiliary, MS quadrupole, and MS source respectively. The mass range was detected between 30 μm to 550 μm, with a scan speed of 1.562 μ/s and the mass spectrometry detector turned on after 5.5 min.

### Quality control

Four quality control (QC) samples were prepared by pooling 20 μL of each sub-aliquoting corresponding sample into a new tube, and MCF derivatization was prepared in the aforementioned manner. The acquisition of a QC spectrum was performed every 15 samples.

### Metabolites identification

The chromatographic characteristics were deconvoluted and identified using Automated Mass Spectral Deconvolution & Identification System software. The metabolites were confirmed by matching both the in-house MFC library spectra >85% and their respective GC retention time being within a 30 seconds window. The identification of remaining putative compounds was used in a commercial NIST mass spectral library.

### GC-MS data mining and normalization

The relative concentration of metabolites was extracted using the MassOmics R-based script through the peak height of the most abundant fragmented iron mass within a predetermined retention time. The background contamination and any carryover from identified metabolites were subtracted by blank samples. To improve quantitative robustness, the relative concentration of identified compounds was first normalized by the internal standard (d4-alanine, d5-phenylalanine, or d5-tryptophan) based on their correlation with metabolites in the QC samples (38). Median centering of QC samples was performed to adjust for the daily batch effects. The dilution correction was achieved by total ion chromatogram (TIC) in urine and plasma, while biomass in weight was applied for tissue and hair samples.

### Absolute quantification of metabolite concentration

Amino acids, fatty acids, and lactic acids were quantified using chemical standards. Levels of these metabolites were first normalized with the appropriate internal standard and then quantified to absolute concentration utilizing calibration curves obtained from the corresponding chemical standard (Five-concentration range from 0~55.4 mM).

### Machine learning development and validation

Since the different machine learning models may predict or rank different classifiers, seven machine learning methods were performed to build the most appropriate binary classification models for various sample types. They were artificial neural network (ANN), decision tree (DT), K nearest neighbor (KNN), logistics regression (LR), naïve bayes (NB), random forest (RF), and support vector machine (SVM). The seven methods were performed using the R package (39–42). The workflow of machine learning *was illustrated in* **Supplementary Figure 1**. After the metabolite dataset was scaled by log_2_ transformation and z-score normalization algorithm, data was randomly split into the training dataset and the testing dataset. The significant features were selected by recursive feature elimination (RFE) methods of the training dataset. Seven supervised machine learning models with the selected features were trained to build efficient classifiers using R-package including Caret, neuralnet, e1071, kknn, and C50 (43–47). To further internally validate the model, a stratified 5-fold cross-validation method was used to tune the hyper-parameter of each model using the testing datasets. The importance ranking of selected features for each machine learning model was used to compute based on feature importance gain (48). Moreover, an independent urine dataset was conducted to further validate the performance of seven machine learning models. The importance ranking features shortlisted from initial models were employed to perform the external validation of the independent dataset for machine learning algorithms. AUC, true positive (TP), true negative (TN), false positive (FP), and false negative (FN) were calculated. The five metrics of machine learning were applied to evaluate the performing algorithm, including accuracy, sensitivity, specificity, positive predictive value, and negative predictive value, which were defined as follows:


Accuracy=(TP+TN)/(TP+FP+TN+FN);

Sensitivity=TP/(TP+FN);

Specificity=TN/(TN+FP);

Positive predictive value=TP/(TP+FP);

Negative predictive value=TN/(TN+FN)

(49).

### Statistical analysis

Non-parametric Kruskal-Wallis with Bonferroni test for *post-hoc* analysis was applied to comparisons of demographics and clinical characteristics between the control group, benign ovarian tumors group, and ovarian cancer group. Chi-square or Fisher’s exact test was used for pairwise comparisons of categorical variables, such as gravidity and parity. Principal component analysis (PCA) was plotted using R program.To adjust for the confounding demographic significantly different in age and BMI of patient, binary logistic regression was conducted to confirm differences in metabolite abundance in the three groups. Then false discovery rates (FDR) were calculated for metabolites by the q-value R package (50). P-value< 0.05 and corresponding FDR< 0.2 was considered statistically significant. UpSet diagram and heatmap were completed by UpSetR and ggplot2 R package respectively (51, 52). The diagnostic ability has been determined by the receiver operating characteristic (ROC) curve. The area under the curve is commonly used to determine the predictability of prediction ability, and a higher AUC value considers the superiority of the classifier. Metabolic pathways were estimated by KEGG metabolic pathways, and chord plots connecting metabolites and their participating metabolic pathways were reconstructed *via* the GOplot R package (53).

## Results

### Clinical characteristics of study participants

Clinical characteristics of patients were shown in **Table 1**. The ovarian cancer group had a significantly lower BMI compared with the control group (25.44 vs 20.40, *p* = 0.002), and the BOT group was significantly younger than the control group (40 vs 54, *p*< 0.000). Gravidity and parity exhibited no significant differences among the three groups. The majority of pathological types of OC was high-grade serous (70.59%), and the proportions of FIGO stage were 29.41%, 11.76%, 52.94%, and 5.88% for I-IV, respectively. Lastly, serum CA125 and HE4 differed between the groups.

**Table 1:** Clinical characteristics of study participants.

Characteristics	Control		Benign ovarian tumors		Ovarian cancer	p-value^e^
		p-value^c^		p-value^d^		
Participants, n (%)	14 (29.79)		16 (34.04)		17 (36.17)	
Age ^a(b)^, years	54 (52.00, 55.75)	0.080	40 (30.25, 48.50)	0.070	48 (46.00, 54.00)	0.001
BMI ^a(b)^, kg/m^2^	25.44 (23.96, 26.60)	0.002	21.95 (20.67, 23.59)	0.443	20.40 (19.96, 23.94)	0.004
Gravidity ^a(b)^	2 (2, 3)	0.677	2 (1.75, 3)	0.398	3 (2, 4)	0.519
Parity ^a(b)^	1 (1, 1)	0.456	1(0,1)	0.983	1 (1, 1)	0.340
FIGO staging (2018)						
I, n (%)	n/a		n/a		5 (29.41)	
II, n (%)	n/a		n/a		2 (11.76)	
III, n (%)	n/a		n/a		9 (52.94)	
IV, n (%)	n/a		n/a		1 (5.88)	
Pathology type						
Hysteromyoma, n (%)	8 (57.14)		n/a		n/a	
Endometrioma, n (%)	2 (14.29)		n/a		n/a	
Uterine prolapse, n (%)	4 (28.57)		n/a		n/a	
Ovarian teratoma, n (%)	n/a		5 (31.25)		n/a	
Chocolate cyst, n (%)	n/a		4 (25.00)		n/a	
Ovarian cyst, n (%)	n/a		6 (37.50)		n/a	
Mucinous cystoadenoma, n (%)	n/a		1 (6.25)		n/a	
High-grade serous ovarian cancer, n (%)	n/a		n/a		12 (70.59)	
Mucinous, n (%)	n/a		n/a		1 (5.88)	
Endometrioid, n (%)	n/a		n/a		1 (5.88)	
Yolk Sac Tumor, n (%)	n/a		n/a		1 (5.88)	
Low-grade serous ovarian cancer, n (%)	n/a		n/a		1 (5.88)	
Papillary serous carcinoma, n (%)	n/a		n/a		1 (5.88)	
CA125 ^a(b)^, U/ml	16.90 (9.05, 27.30)	0.001	23.35 (17.93, 44.88)	0.004	533.70 (156.30, 1000.00)	<0.001
HE4 ^a(b)^, pmol/l	37.60 (35.90, 56.63)	0.022	37.00 (34.85, 43.58)	0.001	164.90 (66.20, 497.70)	0.001

a: median; b: confidence interval (25th percentile, 75th percentile), c: healthy control group compared with ovarian cancer group, p-value adjusted with the Bonferroni method, d: benign ovarian tumors group compared with ovarian cancer group, p-value adjusted with the Bonferroni method, e: Kruskal-Wallis with Dunn post hoc tests for multiple comparisons between the three groups.

n: numbers. n/a: not applicable. CA125: cancer antigen 125. HE4: human epididymis protein 4.

### Metabolomic profiling discrimination in different samples

**Figure 1:**
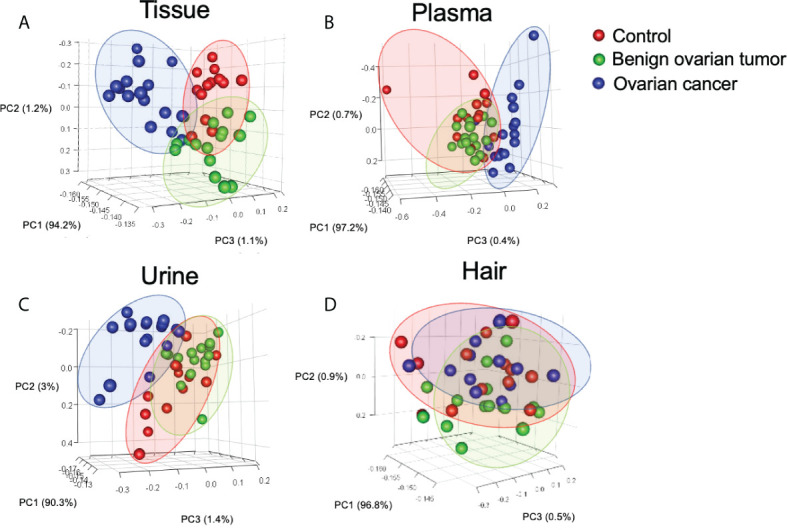
Principal component analysis (PCA) of identified metabolites in tissue **(A)**, urine **(B)**, plasma **(C)**, and hair **(D)** samples. Each point in the plot represents a patient. Red dots represent the control group, and green dots indicate the benign ovarian tumor group, blue dots represent samples derived from the ovarian cancer group. Number of tissue sample in control group (n = 14), BOT group (n = 16), and OC group (n = 16). Number of plasma sample in control group (n = 13), BOT group (n = 16), and OC group (n = 17). Number of urine sample in control group (n = 13), BOT group (n = 15), and OC group (n = 17). Number of hair sample in control group (n = 13), BOT group (n = 16), and OC group (n = 14).

GC-MS based metabolic profiling showed over 150 chromatographic peaks in our study. Among them, a total of 96, 87, 130, and 98 metabolites were identified in the ovarian tissue, plasma, urine, and hair respectively. Unsupervised principal component analysis (PCA) was applied to compare the metabolite compositions (**Figure 1**). PC2 and PC3 were the main components to segregate between OC, control, and BOT groups was observed in ovarian tissue, plasma, and urine samples (**Supplementary Figure 2**). The cancer group was capable of discriminating from control and BOT groups in urine and plasma samples, while no separation was found in the hair sample. The top five metabolites load on each component of ovarian tissue, plasma, and urine samples were shown in (**Supplementary Figure 2**).

### Differential metabolites in study subjects of tissue, urine, plasma, and hair samples

**Figure 2:**
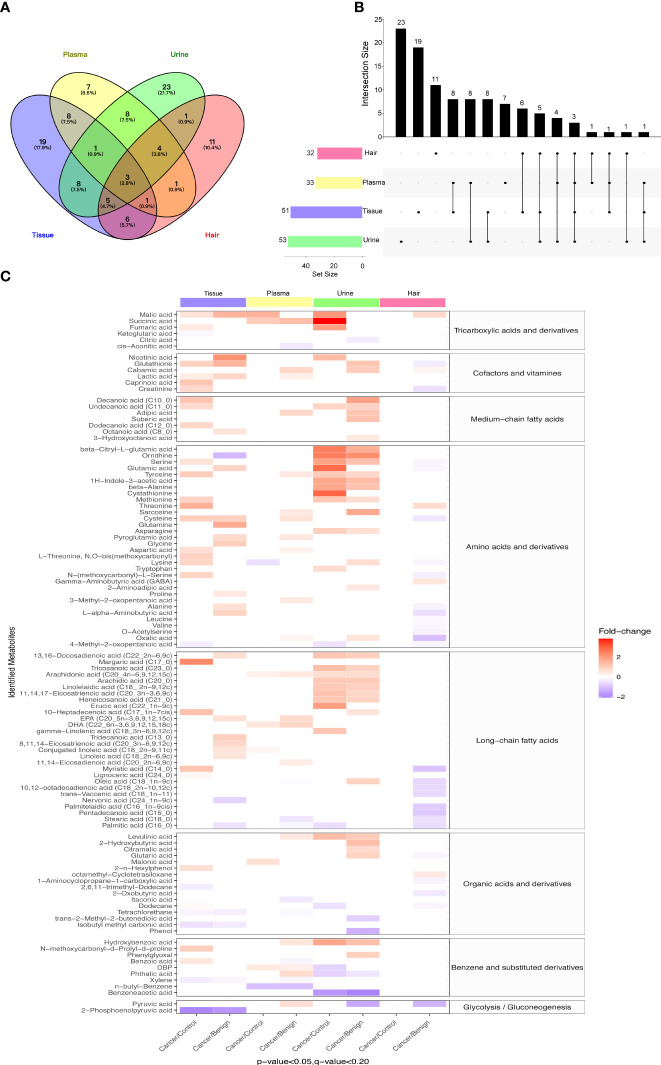
Differential metabolites in study subjects of tissue, urine, plasma, and hair samples. **(A)** Venn diagrams indicated overlapping significant metabolites (p-value < 0.05, FDR < 0.2) in tissue, plasma, urine, and hair samples. **(B)** UpSet plot illustrated the number of different significant metabolites (p-value < 0.05, FDR < 0.2) in tissue, plasma, urine, and hair samples. The individual or connected dots represent the significant metabolite that was either unique to or shared among comparisons in different samples. **(C)** The heatmap showed comparative metabolite profiles of four specimen types and their associated metabolic classification. The relative concentrations of sample metabolites are illustrated *via* log2 scale. Red color blocks represent higher metabolite levels in dividend groups than the divisor groups, whereas blue color blocks represent lower metabolite levels in dividend groups than the divisor groups. Only the metabolites with a p-value (Logistic regression adjusted for gestational age and BMI) less than 0.05 and a q-value (FDR) less than 0.2 are displayed. Number of tissue sample in control group (n = 14), BOT group (n = 16), and OC group (n = 16). Number of plasma sample in control group (n = 13), BOT group (n = 16), and OC group (n = 17). Number of urine sample in control group (n = 13), BOT group (n = 15), and OC group (n = 17). Number of hair sample in control group (n = 13), BOT group (n = 16), and OC group (n = 14).

To eliminate the potential confounding effects of age and BMI, pairwise logistic regressions were performed to compare the metabolite changes between control, BOT, and ovarian cancer in four different types of biological specimens. The Venn map showed that 13 metabolites were statistically different between cancer and non-malignant in both tissue and plasma samples; while urine and hair samples shared 17 and 15 common metabolites with the ovarian tissue samples, respectively (**Figure 2A**). The UpSet plot summarized global overlap metabolites across comparisons for all sample types (**Figure 2B**). Tissue and urine had a higher number of significant metabolites (n = 51, n = 53), and fewer significant metabolites were detected for plasma and hair. Unique significant metabolites for urine, tissue, plasma, and hair were 23, 18, 11, and 7, respectively. Plasma and urine had 16 common significant metabolites, while nine were between plasma and hair. Last, there were only three metabolites in common among the four different sample types.

When comparing ovarian cancer with BOT and control groups for the four different specimen types, a total of 106 metabolites were found to be significantly altered as shown in **Figure 2C** (p< 0.05, FDR< 0.2). The majority of significantly different metabolites among these eight comparisons were TCA cycle intermediates, amino acids and derivatives, fatty acids (long-chain predominantly), cofactors and vitamins, benzene and substituted derivatives, organic acids, and derivatives, and alkanes and derivatives. Most metabolites had a higher relative concentration in the cancer group compared with control or BOT groups for tissue, urine, and plasma samples; while significantly decreased metabolites were observed in the ovarian cancer group in the hair sample. Specifically, except for the hair sample, most of the long-chain fatty acids show an increase in tissue, urine, and plasma. On the other hand, the ovarian cancer group had significantly lower levels of 2-phosphoenolpyruvic acid, 4-Methyl-2-oxopentanoic acid, nervonic acid, stearic acid, and palmitic acid compared to the control group or BOT group.

### Targeted metabolite quantification across different specimen types

**Figure 3:**
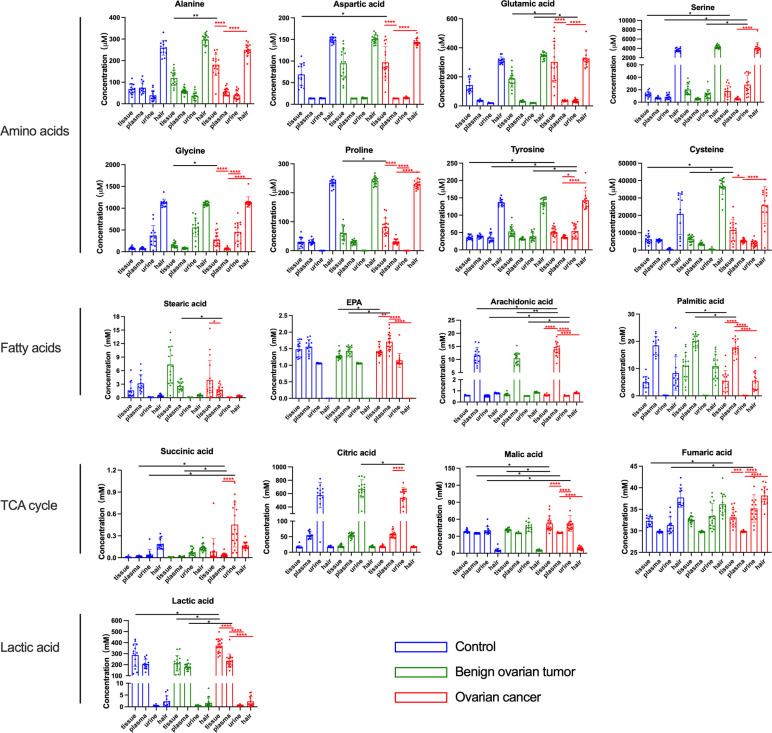
The concentration of amino acids, fatty acids, and lactic acid in the tissue, plasma, urine, and hair samples from control, benign ovarian tumor, and ovarian cancer patients. Blue circles represented metabolites levels collected from control patients, green circles represented metabolites levels collected from benign ovarian tumor patients, and red circles represented metabolites levels collected from ovarian cancer patients. Black asterisks (*) indicated metabolites with significantly different levels between ovarian cancer group vs. control group, and ovarian cancer group vs. benign ovarian tumor group using logistic regression with confounding factors together with a false discovery rate. Red asterisks (*) indicated a significant difference in metabolites between various sample types in the ovarian cancer group using Tukey’s Honest Significant Difference (HSD) test (*p-values< 0.05, **p-values < 0.01, ***p-values < 0.001, ****p-values < 0.0001). Only the significant metabolites with absolute quantification were displayed. Number of tissue sample in control group (n = 14), BOT group (n = 16), and OC group (n = 16). Number of plasma sample in control group (n = 13), BOT group (n = 16), and OC group (n = 17). Number of urine sample in control group (n = 13), BOT group (n = 15), and OC group (n = 17). Number of hair sample in control group (n = 13), BOT group (n = 16), and OC group (n = 14).

To investigate whether the metabolites exhibited distinct profiles in tissue, plasma, urine, and hair samples between OC and non-carcinoma patients, we have conducted absolute quantification of amino acids, fatty acids, TCA cycle intermediates, and glycolytic end-products. Overall, the majority of metabolites showed similar trends in different sample types (**Figure 3**). Particularly, amino acids were reduced in concentration in the magnitude of the ovary, plasma, and urine. Plasma appeared to have the highest fatty acid concentration than the other sample types in OC; while TCA cycle intermediates were the highest concentration in the urine. Lastly, lactic acid expressed the highest concentration in the ovary, and its level was decreased in the plasma and urine samples of the OC group.

### Machine learning algorithms for disease prediction

**Figure 4:**
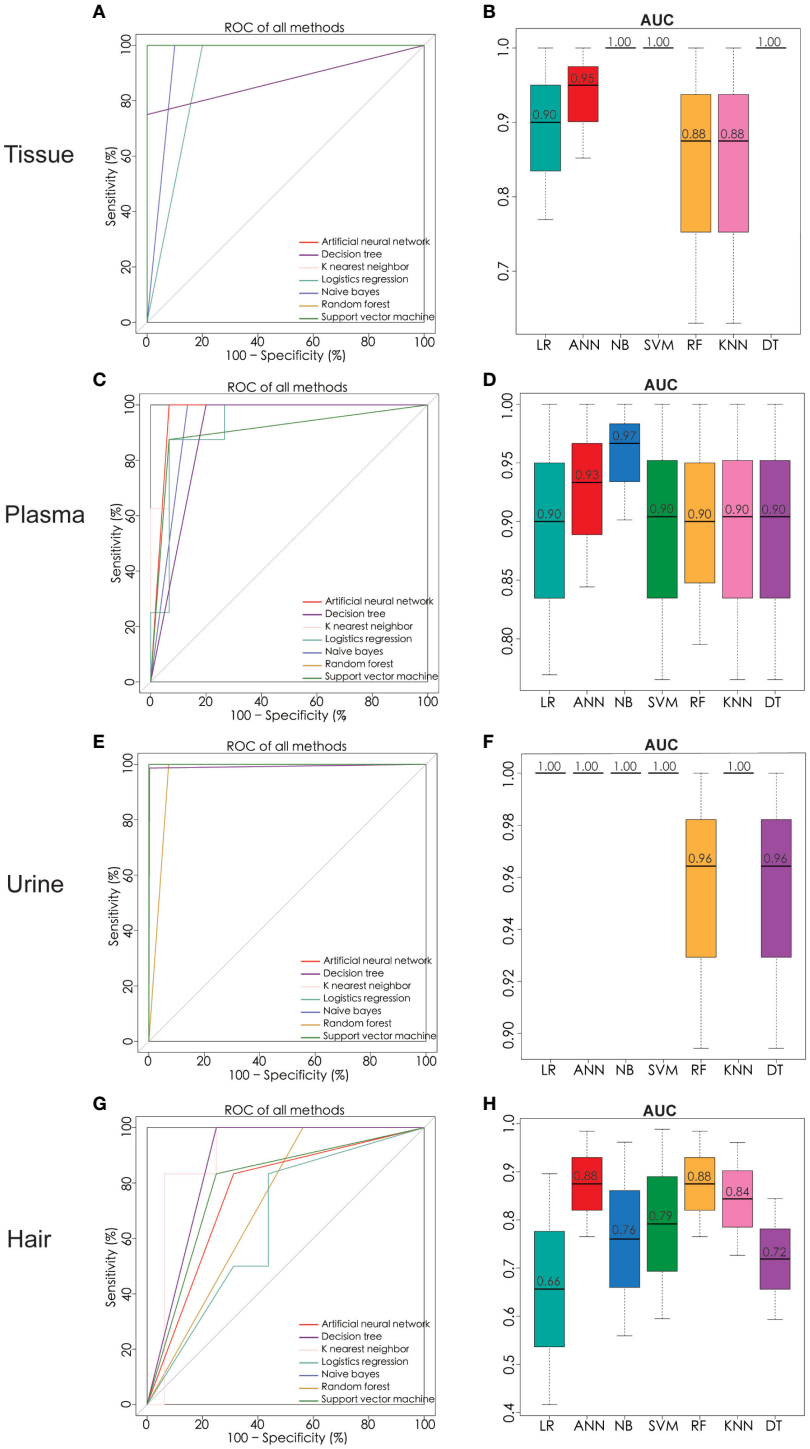
Significant metabolites were nominated and ROC analysis by seven machine learning models of ANN, DT, KNN, LR, NB, RF, and SVM. ROC curve analysis and corresponding AUC (median with 95% confidence interval) of every model on tissue **(A, B)**, plasma **(C, D)**, urine **(E, F)**, and hair **(G, H)**. ROC receiver operating characteristic, AUC area under the ROC curve, ANN artificial neural network, DT decision tree, KNN K nearest neighbor, LR logistics regression, NB naïve bayes, RF random forest, SVM support vector machine. Number of tissue sample in control group (n = 14), BOT group (n = 16), and OC group (n = 16). Number of plasma sample in control group (n = 13), BOT group (n = 16), and OC group (n = 17). Number of urine sample in control group (n = 13), BOT group (n = 15), and OC group (n = 17). Number of hair sample in control group (n = 13), BOT group (n = 16), and OC group (n = 14).

**Figure 5:**
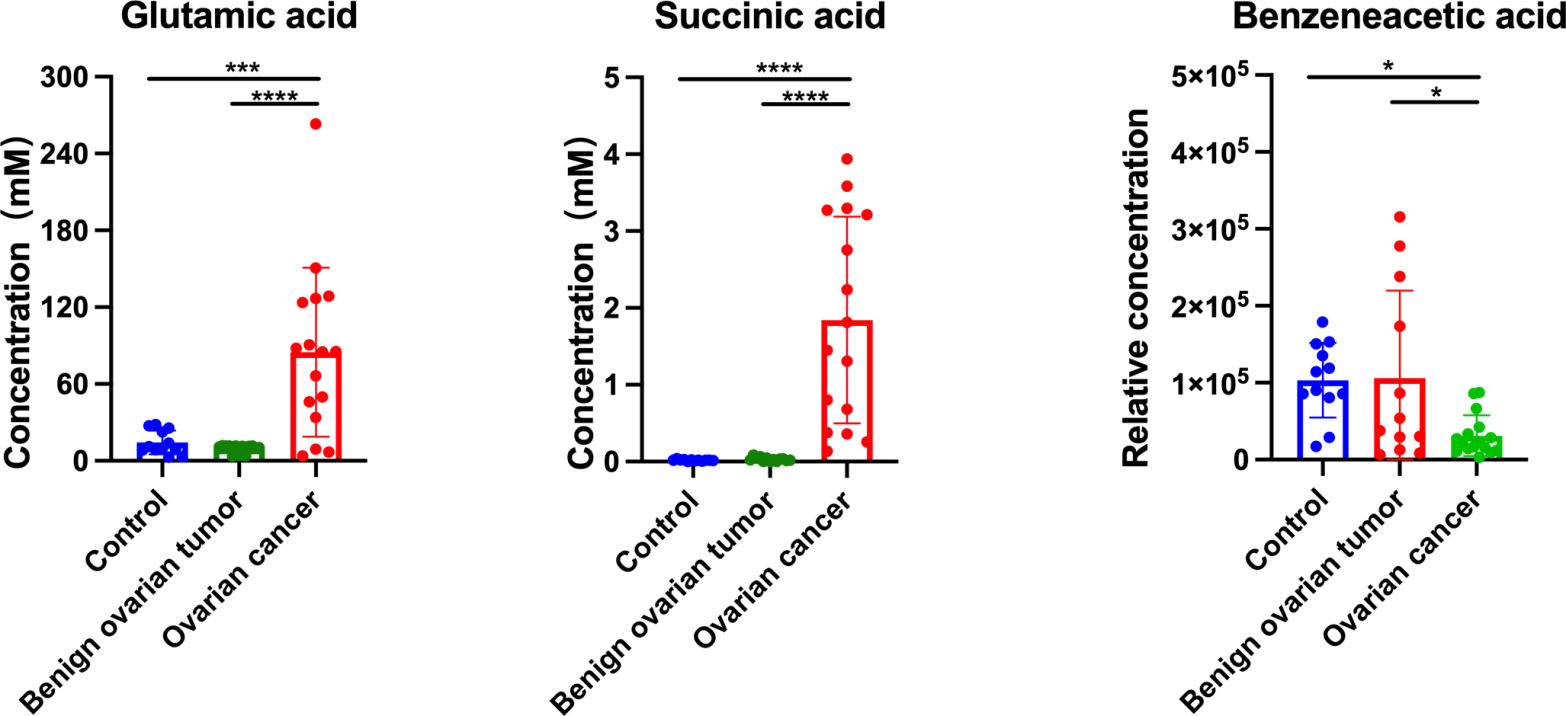
The concentration of glutamic acid, succinic acid, and benzeneacetic acid in the urine sample from control, benign ovarian tumor, and ovarian cancer patients of the validation cohort. Black asterisks (*) indicated metabolites with significantly different levels between ovarian cancer group vs. control group, and ovarian cancer group vs. benign ovarian tumor group using Tukey’s Honest Significant Difference (HSD) test (*p-values < 0.05, ***p-values < 0.001, ****p-values < 0.0001). Number of urine sample in control group (n = 12), BOT group (n = 13), and OC group (n = 16).

In order to determine which metabolites can be used to discriminate different patient groups, the algorithms including artificial neural network (ANN), decision tree (DT), K nearest neighbor (KNN), logistics regression (LR), naïve bayes (NB), random forest (RF), and support vector machine (SVM) were implemented. As a result of the algorithms applied to the classification, a panel of 26 metabolites in tissue, five in plasma, three in urine, and four in hair were nominated. The contributions of nominated metabolites to the performance of each machine learning algorithm (except KNN and NB) were ranked in **Supplementary Figure 3**. The biomarker signatures discriminating the ovarian cancer group from the control and BOT groups are displayed in **Figure 4**. Following internal validation from machine learning models, tissue, urine, and plasma samples showed significant discriminative power (AUC >0.85) except for the hair sample. Particularly, model performances were highly robust for urine (AUC >0.95) based on top-ranking features including succinic acid, glutamic acid, and benzeneacetic acid (**Supplementary Figure 3**), and their concentrations were displayed in **Figure 5**. Specifically, NB (1.0), SVM (1.0), and DT (1.0) were performed the highest AUCs for the tissue sample. NB (0.97) performed the highest AUCs for the plasma sample. LR (1.0), ANN (1.0), NB (1.0), SVM (1.0), and KNN (1.0) performed the highest AUCs for the urine sample. ANN (0.88) and RF (0.88) performed the highest AUCs for the hair sample. Notably, the SVM provided the overall best performance for our dataset because it is a machine learning approach for binary classification and can handle the smaller datasets well (54). RT appeared to give the poorest performance, likely due to underfitting caused by smaller dataset (55). To further assess the performance of the top-ranking features, an independent urine sample was applied to validate the models. We found that the external validation of the independent urine dataset yielded AUC and accuracy over 0.8 (**Supplementary Table 1**). Together with the pros and cons of the different machine learning algorithms (**Supplementary Table 2**), we found that SVM and NB were appropriate models to our relatively small dataset and binary research question.

### Metabolic pathway enrichment analysis

**Figure 6:**
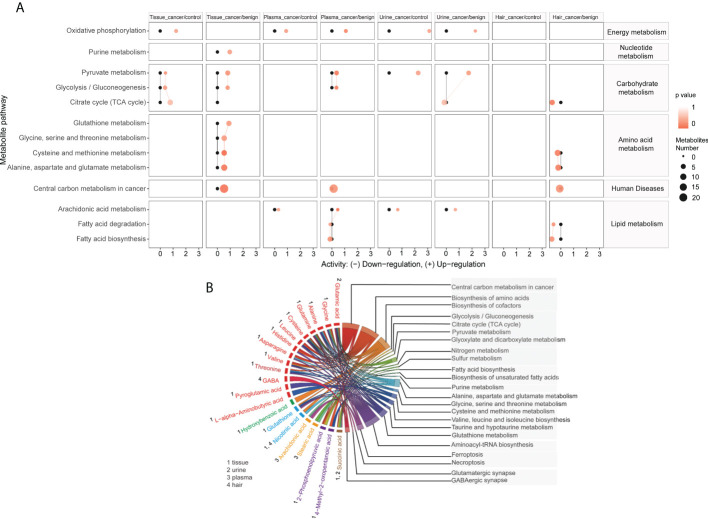
Metabolites are linked to different metabolic pathways. Activities of metabolic pathways in four samples (tissue, plasma, urine, and hair) based on the metabolome of patients. **(A)** Black dots represented metabolic activities in different samples (tissue, plasma, urine, and hair) from control patients that were adjusted to 0. Red dots represented metabolic activities in four samples from ovarian cancer compared to the control or benign ovarian tumor group. The metabolic activities were visualized using the log_2_ scale. The dot size indicated the number of metabolites of the pathway, and the dot color indicated the p-value. A chord plot **(B)** displayed how the metabolites link to different metabolic pathways. Different colors represented different types of metabolites classification (red, amino acids and derivatives, green, benzene and substituted derivatives, blue, cofactors and vitamins, yellow, long-chain fatty acids, Purple, organic acids and derivatives, Burlywood, tricarboxylic acids and derivatives).

To further explore the biological role of identified metabolites, we performed the pathway enrichment analysis based on the KEGG metabolic network (**Figure 6A**). The predicated pathway analysis showed that the metabolism of energy, nucleotides, carbohydrates, and amino acids, were upregulated in cancer tissue. The energy metabolism, carbohydrate metabolism, and lipid metabolism were also upregulated in plasma and urine, while almost all metabolic pathways were downregulated in the hair of OC patients. Then we annotated the KEGG metabolic pathways linked to their shared significant metabolites, shortlisted by seven machine learning models, and illustrated as a chord plot in **Figure 6B**. Half of the metabolites were involved in central carbon metabolism in cancer. The majority of these metabolites were amino acids and cofactors, indicating their potential roles in tumor development. Interestingly, other cancer-related metabolic pathways such as glutathione metabolism, ferroptosis, and necroptosis, were also highlighted. Glutathione metabolism consists of glutamine, glutamic acid, glutathione, cysteine, glycine, and pyroglutamic acid. Ferroptosis encompassed glutamic acid, glutamine, cysteine, glutathione, and arachidonic acid. While another cell death pathway, necroptosis, solely contained arachidonic acid.

## Discussion

Alterations in cellular metabolism have been reported in numerous human cancers and are thought to reflect the metabolic demands related to cancer development. This study performed comprehensive metabolic profiling of ovarian tissue, urine, plasma, and hair samples collected from 47 women diagnosed either with OC or non-carcinoma. Our results identified specific metabolites that could discriminate OC from BOT and control subjects in various samples. OC tissue displayed an elevated concentration of metabolites involved in anaerobic respiration (lactic acid) and antioxidant (glutathione) capability against cell death. Plasma exhibited the most discriminated fatty acid profile between cancer and non-carcinoma groups, while TCA cycle intermediates were most concentrated in OC urine. Only a minor disparity was found. A trend in the opposite direction was found in the hair compared with other sample types. Thus, this study provided novel insights into the metabolic phenotypes of different specimen types that differentiate malignant from benign and normal ovarian pathology.

### Metabolic reprogramming of ovarian cancer tissue

**Figure 7:**
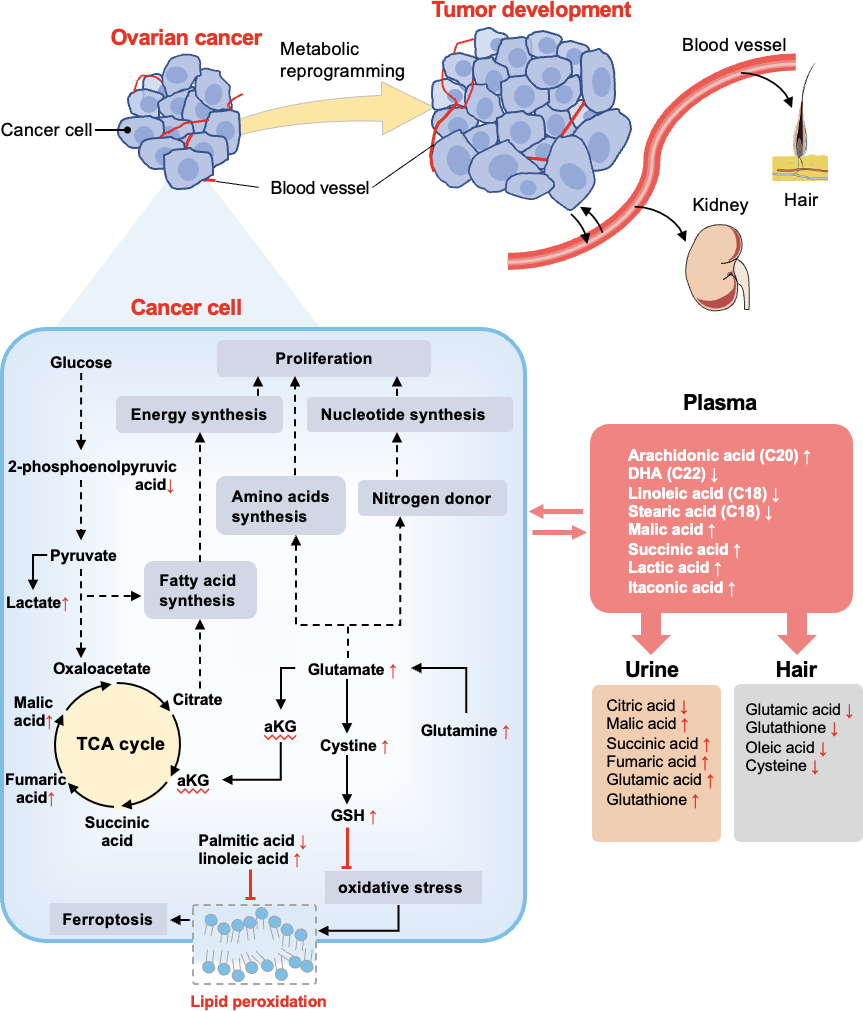
Summary of altered metabolites and metabolic pathways in this study. Reprogramming metabolites of ovarian cancer is thought to support the biological processes that enable tumor development. The metabolome of OC patients and non-malignant patients were compared. In cancer cells, glycolysis, TCA cycle, and glutamate metabolism were upregulated to support fatty acids synthesis, amino acids synthesis, and nucleotide synthesis, and finally contribute to cell proliferation. GSH was conducive to inhibiting oxidative stress, as well as fatty acids assist with inhibiting lipid peroxidation. Vasculature plays an important role in substance exchange with OC tissue and also translates metabolites to the kidney and hair. The red arrows (following the metabolites) indicate increased or decreased regulation of metabolites in OC groups detected in different samples. The black solid arrows depict the movement of metabolites or metabolic reactions, and dashed arrows depict the positive regulatory effects of metabolic components. The red arrows (following the metabolites) indicate increased or decreased regulation of metabolites in OC groups detected in different samples based on significant results in **Figures 2**, **3**.

The global metabolic reprogramming of ovarian cancer could promote nutrients utilization and energy production as well as oxidative resistance (**Figure 7**). We found that glycolytic byproduct (lactate), TCA cycle intermediates (malic acid), and amino acid derivatives (glutamate, cysteine, glutathione) were commonly significantly higher in ovarian cancer patients respectively compared to both control and benign groups; while glycolytic intermediate (2-phosphoenolpyruvic acid) showed the opposite result. The KEGG enrichment analyses also highlighted that energy metabolism, amino acid metabolism, and purine metabolism were upregulated in the cancer group. Several studies also found similar metabolic changes in ovarian cancer. Ha *et al.* reported the general paradigm that the elevated energy metabolism in ovarian cancer cell lines was directly associated with increased lactate levels (24). Denkert *et al.* and Garg *et al.* demonstrated that amino acids and TCA cycle intermediates were elevated in ovarian cancer tissues (56). Based on these findings, we suggested that ovarian cancer promotes anaerobic respiration by breaking down 2-phosphoenolpyruvic acid into lactic acid along with the energy production for addressing the demand for rapid tumor growth (57). Additionally, lactic acid contributes to an acidic microenvironment, which results in cancer cell proliferation and migration *via* enhancing angiogenesis, and decreased cell adherence (58). Through the upregulation of the TCA cycle and amino acids metabolism, large sources of intermediate nutrients are utilized to assemble various molecules. Notably, glutamate plays a key role in the proliferating cells, not only by contributing to amino acid biosynthesis, but also acts as a nitrogen donor for purine and pyrimidine nucleotides biosynthesis (8, 58). Furthermore, some studies have demonstrated that glutamate can fuel the TCA cycle *via* an anaplerotic reaction of the TCA cycle intermediate α-ketoglutarate, which contributes to redox balance and increase NADH production for the proliferation of cancer cells (59, 60). Moreover, buffering oxidative stress is another crucial requirement in cancer progression. In ovarian cancer tissue, the relative concentration of glutathione, linoleic acid, and EPA was increased, while the palmitic acid level was decreased significantly. The metabolic pathway mapping indicated that these significantly changed metabolites were associated with cysteine and methionine metabolism, ferroptosis, and biosynthesis of unsaturated fatty acids pathways. It has been indicated that the concentration of GSH was higher in ovarian cancer tissue than in benign ovarian tumor tissue (61). Several researchers reported that increased GSH often accompanies tumor growth to mitigate the impact of elevated oxidative stress resulting from a rapid metabolic rate (62, 63). GSH not only acts as an antioxidant to minimize oxidative stress but is also profoundly related to cisplatin and carboplatin chemoresistance through reducing drug uptake and increasing drug inactivation in ovarian cancer (64). Moreover, GSH can protect cell death against ferroptosis, a non-apoptotic-regulated cell death culminating with overwhelming lipid peroxidation (65–67). Particularly, MUFAs (oleic acid) can compete with PUFAs (arachidonic acid, linoleic acid) for incorporation into phospholipids to exert anti-ferroptotic effects (68, 69). Yang *et al.* proposed that polyunsaturated fatty acids (PUFAs) are the most susceptible lipids to oxidative damage and could be the potential target for antineoplastic therapy (69). Altogether, the results related to ovarian cancer presented up to now indicate that ovarian cancer undergoes metabolic reprogramming to facilitate energy metabolism and protect against oxidative stress.

### From ovarian tissue to systemic blood

Although tissue biopsy is considered a gold standard for the initial diagnosis, it is neither practical nor desirable for cancer screening. In contrast, other biofluids such as blood, are more convenient specimens for biomarker screening and detecting metabolic dysregulation in circulation. Many metabolomics studies have demonstrated obvious discriminations between ovarian cancer and its counterparts by plasma (18, 29, 56, 70). Consistently, we also observed a clear segregation between ovarian cancer and non-malignant groups using plasma samples. There were five metabolites with an area under the ROC above 0.9 including arachidonic acid, itaconic acid, stearic acid, n-butylbenzene, and malic acid. Arachidonic acid is considered as a pro-inflammatory eicosanoid that modulates tumor cell proliferation and differentiation (71, 72). Chronic inflammation is a well-known risk factor for cancer progression. Jonathan *et al.* also reported that itaconic acid may serve as a crucial regulator for the inflammation response and cytokine production *via* post-translational modifications (73). Apostolov *et al.* found that stearic acid, an omega-6 fatty acid, was related to tumor development *in vivo* (74). Furthermore, blood plays an important role in the provision of nutrients and disposal of metabolic waste products for growing tumors. Interestingly, our targeted analysis indicated that the concentration of long-chain fatty acids was higher in the plasma than in the tissue in ovarian cancer, while an opposite trend was shown for lactic acid. A higher level of fatty acids in plasma could be associated with cancer growth, which could be used to supply important energy sources, maintain the lipid bilayer structure of cancer cells, and transduce oncogenic signals (75, 76). Besides, lactic acid has been reported to be uptaken from blood to the liver and muscles (77). This might explain why blood lactic acid displayed a lower concentration than the tumor tissue. Therefore, blood is a robust specimen to reflect the metabolic alterations of ovarian cancer tissue and many systemic metabolites are associated with inflammation.

### From blood to urine and hair

Urine is another common humoral sample used for clinical screening. In our study, the highest proportion of differential metabolites was found in urine (n=52) compared to plasma (n=32) and hair (n=31) samples. Moreover, three significant urinary metabolites (succinic acid, glutamic acid, and benzeneacetic acid) exhibited the highest specificity and sensitivity of prediction power according to machine learning methods (almost=1).These three significant features also achieved good performance among the seven machine learning algorithms using external validation (**Supplementary Table 1**), pointing out their potential as urinary biomarkers for ovarian cancer. Zhang *et al.* had reported that succinic acid could be used as a urinary biomarker for ovarian cancer (32, 78). Furthermore, Lei *et al.* reviewed that succinic acid facilitates tumor development by providing an energy source and converting tumor-associated M2 macrophages, which suppress the anti-tumor immune response in ovarian cancer (79). By comparing metabolite concentrations between different sample types, succinic acid and citric acid were among the highest concentration metabolites in urine compared to other samples in ovarian cancer patients (**Figure 3**). This is likely due to the renal concentrating mechanism of filtered products from blood (80), which suggested that urinary metabolomic analysis might provide complementary information to plasma metabolomic analysis. Hair is another noninvasive bioanalytical sample that has been used as longitudinal bio-monitors for abnormal conditions such as drug testing and pregnancy complications (81-83). In contrast to other sample types, amino acids and fatty acids were significantly downregulated in the ovarian cancer group than control and BOT groups. We speculate that fewer blood metabolites assimilated into hair are due to the high metabolic demand by tumor tissue. Further research is needed to understand this phenomenon. In our study, however, the result of PCA analysis could not stratify ovarian cancer patients from control and BOT patients (**Figure 1D**), and the classification model suggested a poor predictive effect of metabolites (**Figures 4G, H**). Thus, hair is not a suitable sample choice for screening ovarian cancer.

Despite the promising findings, our study has several limitations. The dietary questionnaire should be implemented for studied participants. The sample size was relatively small for machine learning approaches, and more samples are needed to further validate the reliability and prediction capability of plasma and urine as a biospecimen for biomarker discovery in ovarian cancer. Cell and animal studies should be performed to further validate the metabolic mechanisms of ovarian cancer across different tissue types.

## Conclusion

In summary, this is the first metabolomic study attempt to track the metabolic changes between different specimen types of ovarian cancer. Metabolic reprogramming of ovarian carcinoma was mainly characterized by promoting tumor energy metabolism and protecting against oxidative stress. Blood was related to inflammatory response, while TCA cycle intermediates were concentrated in the urine. We have shortlisted several metabolites in plasma and urine as an initial hint for potential biomarkers in ovarian cancer. Altogether, the study provides important knowledge on the metabolic characteristics of differential biological specimens to reveal global metabolic changes in response to ovarian cancer.

## Data availability statement

The datasets presented in this study can be found in online repositories. The names of the repository/repositories and accession number(s) can be found in the article/**Supplementary Material**.

## Ethics statement

The studies involving human participants were reviewed and approved by the Research Ethics Committee of the Second Affiliated Hospital of Chongqing Medical University, China (202164). The patients/participants provided their written informed consent to participate in this study.

## Author contributions

XZ contributed to sample and data collection, performed the statistical analysis, interpreted the results, and wrote the manuscript. RR and SG commented on the design and revised the manuscript. MS and XS contributed to sample collection. FL was responsible for the machine learning algorithms analysis. YZ, XT, and YY devised the original laboratory study and interpreted the results. AL and WH supported the writing of the manuscript and directed the project. TY and TH are the guarantors of this work and, as such, have full access to all the data in the study and take responsibility for the integrity of the data and the accuracy of the data analysis. All authors contributed to the article and approved the submitted version.

## Funding

This work was supported by the National Natural Science Foundation of China (No.81871185), Chongqing Municipal Education Commission (KJZD-K202100407), Chongqing Science & Technology Commission (cstc2021jcyj-msxmX0213), Kuanren Talents Programs of the Second Affiliated Hospital of Chongqing Medical University.

## Acknowledgments

We are grateful to the patients and clinical staff (Xiaojing Dong, Xingwei Jiang, Xiaolin Gan, and Xiaojiao Li) from the department of obstetrics and gynecology department (The Second Affiliated Hospital of Chongqing Medical University, Chongqing, China) for their generous contributions to this study.

## Conflict of interest

The authors declare that the research was conducted in the absence of any commercial or financial relationships that could be construed as a potential conflict of interest.

## Publisher’s note

All claims expressed in this article are solely those of the authors and do not necessarily represent those of their affiliated organizations, or those of the publisher, the editors and the reviewers. Any product that may be evaluated in this article, or claim that may be made by its manufacturer, is not guaranteed or endorsed by the publisher.
