# Supplementary figures and images for: Complex metabolic interactions between ovary, plasma, urine, and hair in ovarian cancer
Source: Front Oncol. 2022 Aug 2;12:916375. doi: 10.3389/fonc.2022.916375 (PMC9379488; doi:10.3389/fonc.2022.916375)

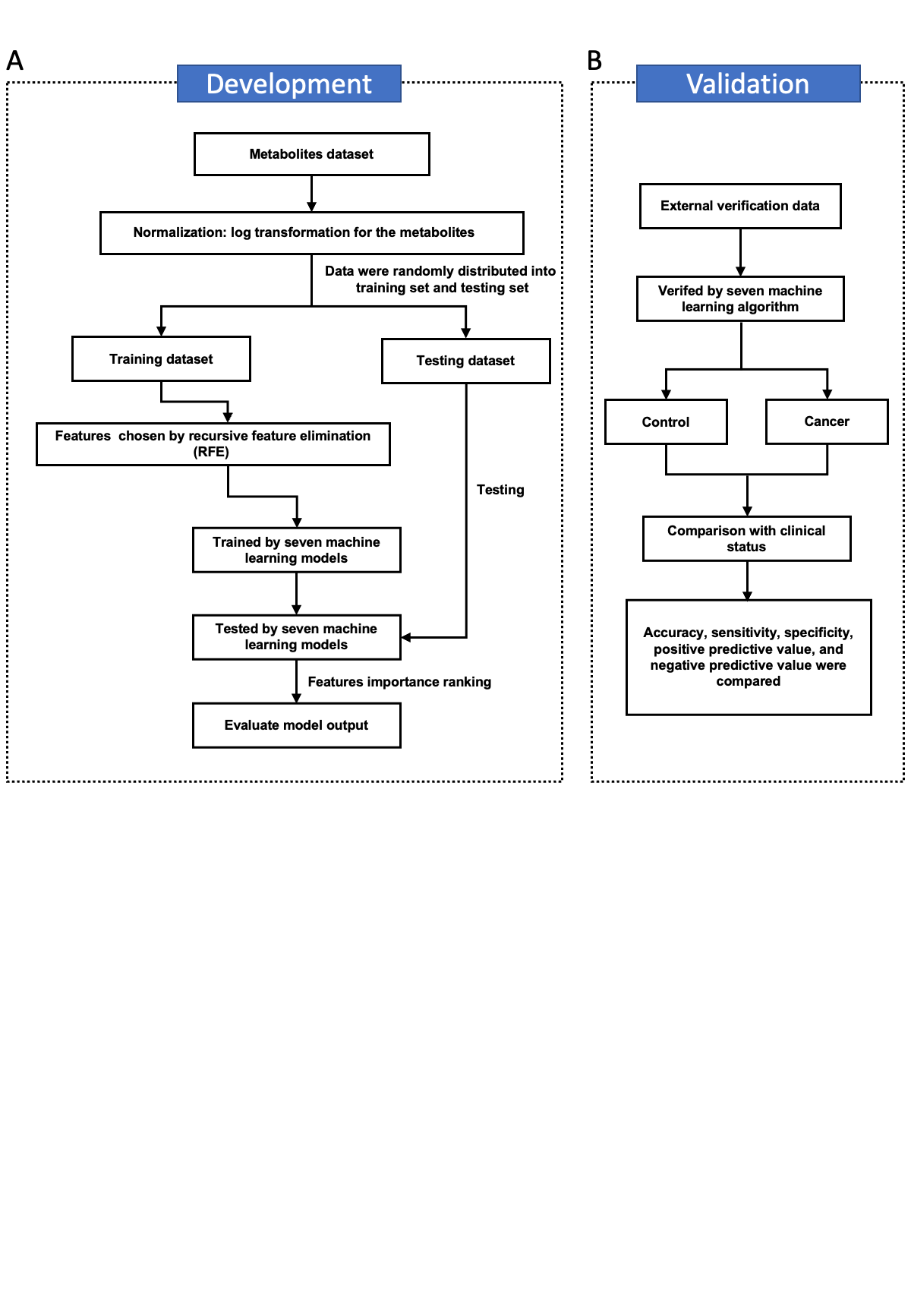

Supplement: Supplementary file 1 [file Image_1.tiff]

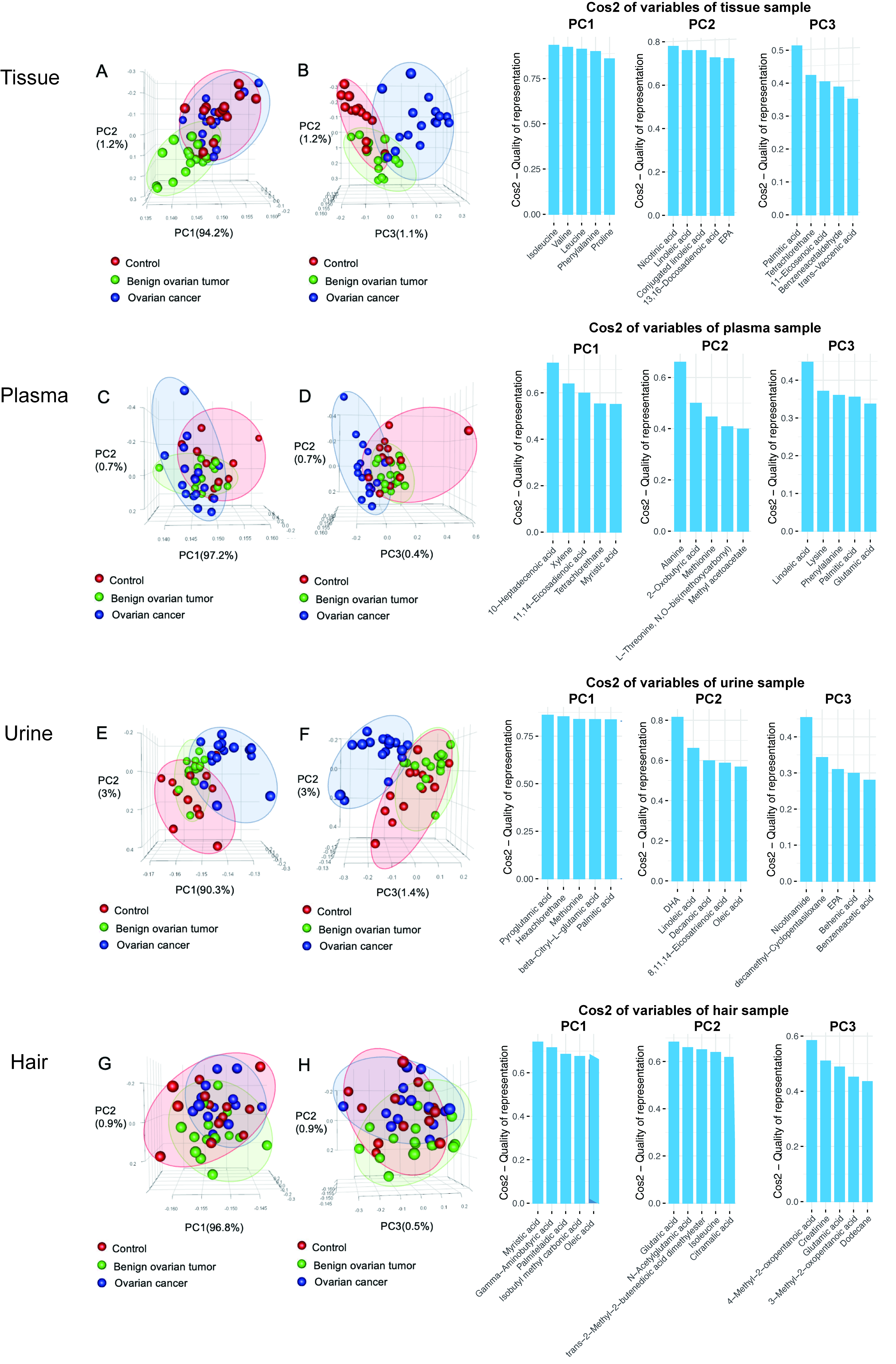

Supplement: Supplementary file 2 [file Image_2.tif]

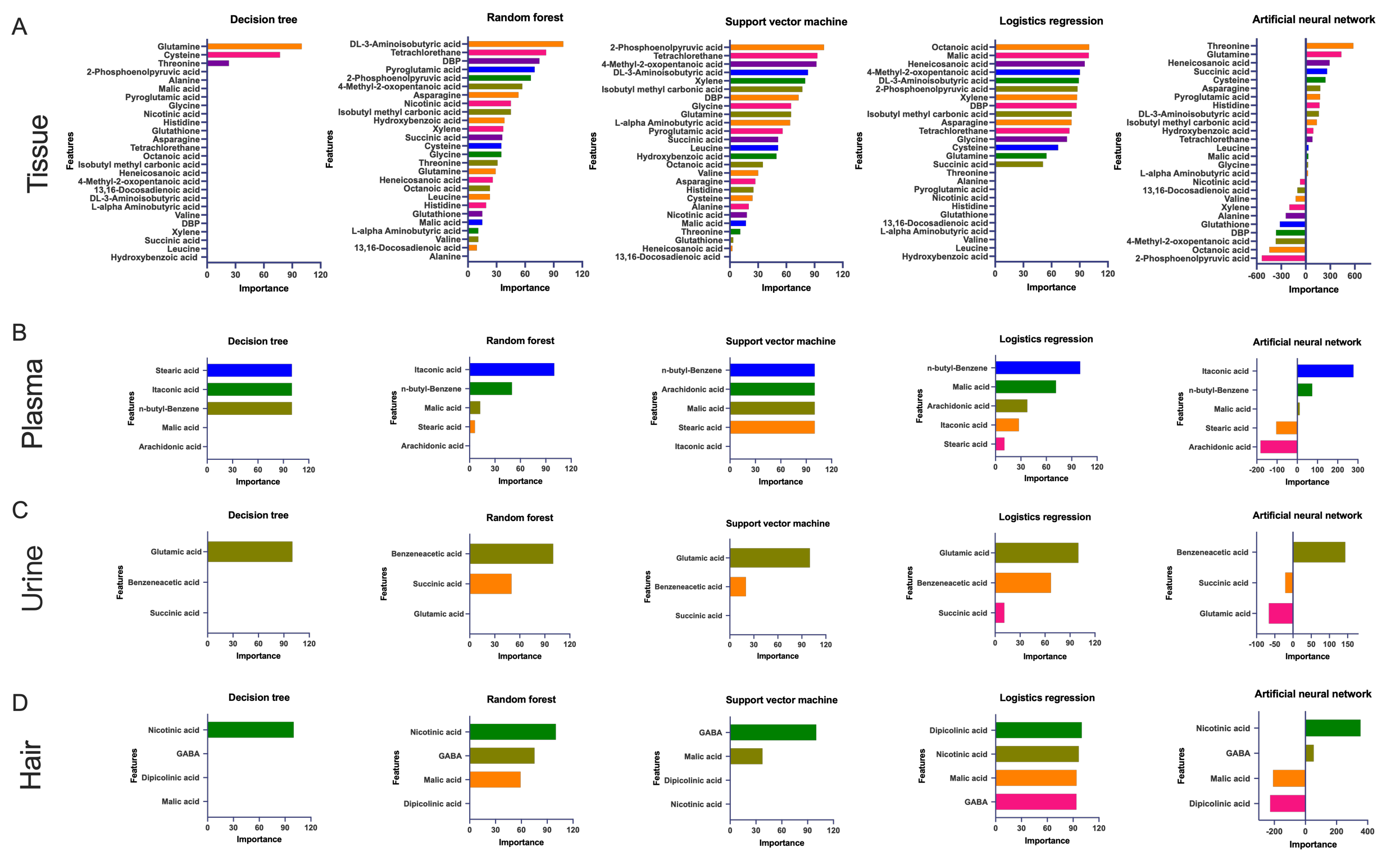

Supplement: Supplementary file 3 [file Image_3.tiff]
