# Supplementary material for: Complex metabolic interactions between ovary, plasma, urine, and hair in ovarian cancer
Source: Front Oncol. 2022 Aug 2;12:916375. doi: 10.3389/fonc.2022.916375 (PMC9379488; doi:10.3389/fonc.2022.916375)
Supplement: Supplementary file 4 [file Table_1.docx]

**Supplementary Table 1 Validation of machine learning algorithms using an independent dataset for the urine sample**

|  | Algorithms | | | | | | |
| --- | --- | --- | --- | --- | --- | --- | --- |
|  | LR | RF | DT | ANN | SVM | NB | KNN |
| AUC | 0.90 | 0.98 | 0.83 | 0.99 | 0.82 | 0.94 | 0.92 |
| Accuracy | 0.88 | 0.85 | 0.83 | 0.90 | 0.85 | 0.90 | 0.93 |
| Sensitivity | 0.96 | 0.88 | 0.84 | 0.96 | 0.96 | 0.96 | 0.96 |
| Specificity | 0.75 | 0.81 | 0.81 | 0.81 | 0.69 | 0.81 | 0.88 |
| PPV | 0.86 | 0.88 | 0.88 | 0.89 | 0.83 | 0.89 | 0.92 |
| NPV | 0.92 | 0.81 | 0.76 | 0.93 | 0.92 | 0.93 | 0.93 |

Sensitivity = TP / (TP + FN); Specificity = TN / (TN + FP);

Positive predictive value = TP / (TP + FP); Negative predictive value = TN / (TN + FN).

ANN, artificial neural network; DT, decision tree; KNN, K nearest neighbor; LR, logistics regression; NB, naïve bayes; RF, random forest; SVM, support vector machine; TP, true positive; TN, true negative; FP, false positive; FN, false negative; PPV, positive predictive value; NPV, negative predictive value.
