# Supplementary material for: Complex metabolic interactions between ovary, plasma, urine, and hair in ovarian cancer
Source: Front Oncol. 2022 Aug 2;12:916375. doi: 10.3389/fonc.2022.916375 (PMC9379488; doi:10.3389/fonc.2022.916375)
Supplement: Supplementary file 5 [file Table_2.docx]

Supplementary Table 2 The pros and cons of the seven machine learning methods

| Algorithms | Advantages | Disadvantages |
| --- | --- | --- |
| Artificial neural network (1) | - Identify complicated nonlinear interactions between dependent and independent variables. - Efficiently capture classification problems. | - Researchers cannot witness the precise decision-making process due to a black box within. - The predictor independent variables must be pre-processed. |
| Decision tree (2,3) | - Suitable for logics and classifying data. - Easy to comprehend and understand. | - The risk of producing overfitted. - lower accuracy compared with other models. |
| K-nearest neighbor (4) | - Does not require the usage of probability values. - Simple algorithm and can classify quickly. | - All traits are given the same weight, which might lead to unsatisfactory classification results. - No ranking was offered as to which features are most important in contributing to the suitable categorization. |
| Logistics regression (4) | - A powerful and well-established approach for modeling a dichotomous variable. | - Ignores the linear relationship between variables. - May overestimate the prediction performance owing to sampling bias. |
| Naïve  Bayes (1,5) | - Suitable for binary and multi-class categorization. - Requires less amount of training data. | - Classes must mutually exclude one another. - The presence of dependency has a detrimental impact on classification performance. - Cannot offer the importance ranking of the features. |
| Random forest (2) | - A few parameters to be adjusted. - Can handle high dimensional data. | - More complicated and time-consuming. - The number of basic classifiers must be established. - Less suitable for regression. |
| Support vector machine (6) | - Robust at the binary classification. - Has a lower chance of overfitting. - Requires less amount of training data. | - Cannot classify more than two classes for generic SVM. - Does not perform well if the data contains noise. - Difficult for scaling large datasets. |

**Reference**

1. Ahn JC, Connell A, Simonetto DA, Hughes C, Shah VH. Application of Artificial Intelligence for the Diagnosis and Treatment of Liver Diseases. *Hepatology* (2021) 73:2546–2563. doi: 10.1002/hep.31603

2. Iddamalgoda L, Das PS, Aponso A, Sundararajan VS, Suravajhala P, Valadi JK. Data Mining and Pattern Recognition Models for Identifying Inherited Diseases: Challenges and Implications. *Front Genet* (2016) 7: doi: 10.3389/fgene.2016.00136

3. Wiemer JC, Prokudin A. Bioinformatics in proteomics: application, terminology, and pitfalls. *Pathol - Res Pract* (2004) 200:173–178. doi: 10.1016/j.prp.2004.01.012

4. Uddin S, Khan A, Hossain ME, Moni MA. Comparing different supervised machine learning algorithms for disease prediction. *BMC Med Inform Decis Mak* (2019) 19:281. doi: 10.1186/s12911-019-1004-8

5. Zhang Z. Naïve Bayes classification in R. *Ann Transl Med* (2016) 4:241–241. doi: 10.21037/atm.2016.03.38

6. Damiati SA. Digital Pharmaceutical Sciences. *AAPS PharmSciTech* (2020) 21:206. doi: 10.1208/s12249-020-01747-4
